# Digital Transformation in Health Care: Are We on the Right Track?

**DOI:** 10.2196/90380

**Published:** 2026-07-21

**Authors:** Guilherme Tortorella, Flavio Fogliatto, Roberto Vassolo, Alejandro Mac Cawley, Diego Tlapa

**Affiliations:** 1 Department of Mechanical Engineering, The University of Melbourne, 2204/915 Collins St, Docklands, Melbourne, Australia, 61 492481774, 61 492481774; 2 IAE Business School, Austral University, Buenos Aires, Buenos Aires F.D, Argentina; 3 Business School, Fundação Dom Cabral, Belo Horizonte, Minas Gerais, Brazil; 4 Industrial Engineering Department, Universidade Federal do Rio Grande do Sul, Porto Alegre, Rio Grande do Sul, Brazil; 5 Department of Industrial and Systems Engineering, Pontificia Universidad Católica de Chile, Santiago, Santiago Metropolitan, Chile; 6 Center for Advanced Transportation Logistics, and Economic Competitiveness (CATLEC), Santiago, Chile; 7 Centro de Investigación e Innovación VitiScience, Santiago, Chile; 8 Industrial Engineering Department, Universidad Autónoma de Baja California, Ensenada, Baja California, Mexico

**Keywords:** digital transformation, health care, Healthcare 4.0, Industry 4.0, sociotechnical systems

## Abstract

Health care digital transformation is gaining increasing attention, despite the observed challenges in its implementation. The envisioned benefits, together with the growing need for better health care, are motivating academia, organizations, regulatory agencies, and governments to develop more effective digital health care solutions. Through extensive debates among the authors, this paper discusses how digital transformation is being conducted in the health care sector. Our discussion relies on concepts from sociotechnical systems theory, categorizing it into 3 social (people, culture, and goals) and 3 technical (processes/procedures, infrastructure, and technology) dimensions. Drawing on both the literature discussed in this paper and our combined academic and practical experience—each author contributing over a decade of work in health care digital transformation—we examined current digitalization efforts from a sociotechnical standpoint. Overall, we argue that both social and technical dimensions present elements that have either encouraged or discouraged the progress of health care digital transformation. The identification of current trends on such (on- and off-track) elements allowed the formulation of 12 propositions across each sociotechnical dimension for future testing and validation. This approach can help establish better government policies, foster private initiatives, and shift regulatory guidelines to support a successful digital transformation in health systems. Finally, from a research perspective, we outline some opportunities for further interdisciplinary investigation in the field, promoting advances in the understanding of health care digital transformation.

## Introduction

Health care systems comprise all organizations, individuals, and activities aimed at promoting, restoring, or maintaining health [1], including efforts to influence health determinants and implement improvement initiatives [2]. Despite this broad scope, many descriptions of health care systems adopt a reductionist standpoint [3,4], neglecting the interrelationships among components and essential functions, such as responsiveness to legitimate expectations, respect for dignity, fair financing, service provision, and enabling functions (eg, stewardship, financing, and resource generation) [5]. This conceptual diversity has led to variations in health care systems worldwide, shaped by regional needs and resources [6]. While health care system performance in some countries is adequate, many still struggle with access, quality, and equity [7], highlighting the need for systemic improvements across processes and organizations [8].

The pursuit of improved quality and efficiency has long motivated the integration of technologies into health care [9,10]. In the 1960s, computers were first applied to standardize and share medical data [11]. During the late 1970s, personal computers introduced new technological solutions to the health care sector [12]. For example, the early electronic medical record (EMRs) initiatives demonstrated how computers could store and retrieve patient data more efficiently, improving record accuracy and accessibility. The advent of the internet in the 1990s further expanded the role of technology in health care [13], such as the use of online medical information platforms, allowing patients to access reliable health information without needing to visit a provider. More recently, the advances of the Fourth Industrial Revolution have intensified digitalization efforts aimed at creating more efficient therapeutic structures and support processes [14,15]. For instance, artificial intelligence (AI) tools have been applied in various ways in health care, from providing 24/7 patient support, symptom checking, and appointment management [16] to assisting with medical imaging and diagnostics [17]. These advances have been enabled by the availability of more effective and affordable technologies, miniaturized sensors with greater portability, and enhanced data acquisition and management capacities [18-20].

The digital transformation of health care continues to face substantial barriers. Beyond technological constraints, political and economic interests, as well as pressures from organizations, associations, and lobbyists, hinder effective implementation [21,22]. Progress has also been uneven across countries and over time [23-25], raising concerns about the consistency and sustainability of digitalization efforts. Given that digital transformation is widely viewed as a key driver of competitiveness and resilience in health care systems [26-28], these barriers highlight the need for a more systematic and sector-specific approach.

This paper presents a viewpoint about the digitalization of health care through debate, advocacy, and refutation [29]. To frame the discussion, we adopt the sociotechnical systems (STS) theory, which emphasizes the joint optimization of social and technical subsystems [30-32]. Literature focusing on the integration of digital technologies into hospitals and clinics also provided the basis for our discussion. This led to the formulation of research propositions (RPs) according to each STS dimension, which serves as guidance for future testing and validation.

The paper contributes by offering a structured examination of health care digital transformation from both theoretical and practical perspectives, identifying elements that may favor (ie, on-track elements for a successful health care digital transformation) or hinder (ie, off-track elements for a successful health care digital transformation) its effective implementation. It highlights key sociotechnical factors shaping digitalization and outlines future research opportunities to advance more effective and resilient health care systems. These outcomes may be useful for different stakeholders, such as health care practitioners handling digital transformation, academics whose research focuses on health care digitalization, and policymakers whose activities are impacted by novel advances in digital technologies.

## Health Care Digital Transformation

Digital transformation is the process of incorporating technologies into processes, products, and services to change how organizations operate, deliver value, and adapt to evolving needs [33,34]. It is also about redesigning processes, culture, and experiences to improve efficiency and agility while creating opportunities for growth and innovation [35,36]. Health care digitalization has been distinctly termed as e-Health [37], Health 4.0 [38], Healthcare 4.0 [39], Care 4.0 [40], and Smart Health [41]. It has also been classified into four categories [42]: (1) diagnosis, (2) treatment, (3) follow-up, and (4) supply chain. Despite differences in nomenclature [43], all these approaches similarly foster health care digitalization, advancing treatments, administrative processes, and the supply chain [44,45]. New technologies improve treatments in the short term and yield gradual gains in administrative processes over time [46]. Health care digital transformation aims to create a more patient-centric care system [9,47], though its success depends on multiple factors. Capital investment, skilled labor, and the socioeconomic context influence digitalization capacity [48,49]. Clinical infrastructure and data regulation are also critical enablers [50]. Technologies include hardware and software to support storage, sharing, and use of health care information for communication and decision-making [51,52]. These advances can enhance patient welfare, staff motivation, and productivity while reducing errors, delays, and costs [53-55]. For instance, AI has been commonly used to monitor patient vitals in real time, providing proactive health insights and standing out as a disruptive technology in the health care setting [56].

Another potential contribution of digital transformation is related to preventive health care, which reduces the incidence and impact of disease by focusing on early detection, risk reduction, and health promotion [57]. Digitalization strengthens this approach by enabling continuous health monitoring through wearables, mobile apps, and connected devices, generating real-time data on behaviors and physiological conditions [58]. When combined with electronic health records (EHRs) and advanced analytics, these data allow health care providers to identify risk patterns, predict potential conditions, and intervene earlier with personalized strategies [59]. This shift moves health care from a reactive, treatment-focused model to a proactive system centered on maintaining health and preventing illness. Moreover, digitalization enhances patient engagement and supports population health management [60]. Telemedicine, patient portals, and digital platforms empower individuals to access information, communicate with providers, and adopt healthier behaviors through personalized guidance and reminders [61]. At a broader level, aggregated data help organizations and policymakers detect trends, target high-risk populations, and design more effective preventive interventions [62].

The World Health Organization’s (WHO) Global Strategy on Digital Health focuses on strengthening health systems by applying digital technologies that empower patients and promote the vision of health for all [63]. Digital health care strategies encompass a wide range of tools, including EHRs, telemedicine, mobile health, wearables, and data analytics. Therefore, national digital health strategies are essential to guiding governments’ efforts in this domain [64]. In Europe, most countries have national digital health strategies or related policies, though with heterogeneous approaches [65]. A notable regional initiative is the Cross-Border eHealth Information Services, enabling secure and interoperable exchange of prescriptions and patient summaries among European countries [66]. Estonia exemplifies advanced integration with a countrywide digital record for all patients and progress toward personalized medicine through genomics [67], which contrasts with the results observed in Australia. Generally, wealthier countries tend to achieve higher adoption rates, such as Canada, where nearly 90% of clinicians use EMRs [68]. In Latin America, EMRs are legally regulated in 16 of 21 countries, though adoption varies significantly between public and private sectors [69]. In Mexico, up to 45% of private professionals conduct remote consultations and 43% use EMRs [70]. Across Africa, digital health varies widely; most sub-Saharan countries face fragmentation, limited scalability, and unequal investment [71,72].

Despite the differences in digitalization initiatives and health care application contexts (Table 1), it is noteworthy that health care digitalization has been a key strategy in large economies. Efforts on how to improve, standardize, and ensure the ethical handling of electronic medical data records have been commonly observed across these countries [7,73]. Additionally, France, Brazil, and Australia are focused on expanding telemedicine (or telehealth) to improve people’s access and reduce the overload on existing health care infrastructure [74,75].

**Table 1.** Digital strategies for the 15 largest economies.

Country	Digital strategy	Description/characteristics
United States	Federal Health Information Technology Strategic Plan [76]	Overarching framework for the US digital health policy EHI^a^, Interoperability, and AI tools
China	Healthy China 2030 [72]	EHR^b^ and EMR^c^ for every citizen National population health information platform
Germany	Digitalization Strategy for Health and Care (Digital Together) [77]	National digital health record, EPR^d^, digital health apps, and AI^e^ in diagnostics and innovation
India	National Digital Health Blueprint [78]	Digital health ecosystem to improve efficiency and effectiveness
Japan	Japan Vision: Health Care 2035 [7]	PHR^f^ and national platform, interoperability and data sharing, telemedicine, AI in diagnostics, and health tech innovation
United Kingdom	Digitize, connect, transform A plan for digital health and social care [73]	Digital inclusion, EPR, NHS^g^ app, AI and genomics integration, and federated data platform
France	Digital Health Strategy 2030 [65] My Health 2022	National digital health innovation and transformation architecture EHR, telemedicine, interoperability and data sharing, AI, and innovation
Italy	New Health Information System [79] EHR 2.0 National telemedicine platform	Standardizes data collection across regions to monitor and evaluate the National Health Service Provides secure access to personal health data Platform enabling televisits, telemonitoring, and teleconsultations
Canada	Pan-Canadian Health Data Strategy [80]	To create a unified, interoperable health data ecosystem for better care, research, and public health decision-making
Brazil	National Health Data Network (RNDS) [81] Digital Health for Brazil [75]	Health databases integration, EHR system, interoperability, telemedicine, mHealth app, unified digital platform, AI, and big data
Russia	Strategic Direction in the Field of Digital Transformation of Healthcare [82]	Digital health services, AI and digital twins, and the use of domestic technology
Spain	Digital Health Strategy of the National Health System [83]	Promotes digital health services, interoperability, and health data analysis Health card, electronic prescription, digital medical record, and AI integration
Korea	Health Technology Reassessment [84]	System to manage the life cycle of health technologies
Australia	National Digital Health Strategy [74]	My Health Record Electronic prescribing Virtual care and telehealth expansion
Mexico	National Digital Strategy [85]	Broad strategy on governance, infrastructure, and social policy, covering universal and effective health

a EHI: electronic health information.

b EHR: electronic health record.

c EMR: electronic medical record.

d EPR: electronic patient record.

e AI: artificial intelligence.

f PHR: personal health record.

g NHS: National Health Service.

Despite well-documented benefits and available technologies, health care digitalization has traditionally faced resistance [86]. Kassirer [87] reported the unpreparedness of physicians to integrate the internet into medical practice in the 1990s, and later studies showed concerns about the negative implications of technology adoption on physicians’ examination and diagnostic skills [88]. Resistance among health care workers may derive from the disruption of routines, generational differences, or limited competencies [89-91]. These findings highlight the importance of addressing the social dimensions of digitalization, which emphasize the joint optimization of social and technical elements [92].

## STS Theory and Digital Transformation

STS theory is based on 2 core principles. First, the interaction between social and technical aspects determines organizational performance [93]. Such interaction includes both linear and nonlinear relationships, intentional or emergent [94]. Second, optimizing social or technical aspects alone tends to generate unintended or counterproductive outcomes [31,95]. Consequently, STS theory fosters joint optimization, in which the design of social and technical subsystems is concurrently conducted [30]. Such integration enables new possibilities for work and supports technological change [32].

A widely used model for assessing STS is the Leavitt [96] framework, later refined by several researchers [97-99]. Originally encompassing 4 dimensions (people, task, structure, and technologies), it evolved to six interrelated dimensions: (1) people, (2) culture, (3) goals, (4) processes/procedures, (5) infrastructure, and (6) technology [100,101]. These dimensions provide a systemic structure for analyzing complex STS, such as health care.

Given that digital transformation integrates new technologies and relies on human-technology interaction, it can be viewed as a sociotechnical process [34,102]. Successful implementation requires a holistic organizational approach that simultaneously addresses human, structural, and technological dimensions [91]. Many studies have adopted STS theory to present complementary findings. For instance, Sony and Naik [22] proposed an STS-based framework for designing and integrating digital technologies. Imran et al [103] demonstrated that leadership, structures, and culture are critical enablers of digital transformation. Thomas [104] identified the key drivers of knowledge management in this context, while Iden and Bygstad [105] emphasized the need for the joint consideration of social and technical factors to facilitate digital transformation. Overall, the literature indicates that STS theory offers a robust theoretical foundation for understanding and guiding digital transformation in health care.

## Methodological Approach

The topic was examined through multiple perspectives and by drawing on existing scholarship to encourage critical reflection and support informed discussion. Although partly grounded in original research, discussion papers typically situate their analysis within the broader body of literature, addressing issues in a comprehensive and balanced manner [106]. Such work is commonly found in academic, organizational, and policy-related settings. Generally, the interpretations and conclusions presented in discussion papers reflect the viewpoints of their respective authors [107-109]. In this sense, discussion papers function as a basis for reasoned debate and decision-making, offering a structured way to explore complex issues, fostering stakeholder engagement, and stimulating the development of new ideas and solutions [110].

Accordingly, using both advocacy and counterargument approaches [29], we discussed cases of health care digital transformation at different stages of maturity and the strategies adopted in each context. To conceptually address the challenges of digital transformation in health care and to differentiate initiatives progressing effectively from those that are not, we adopted STS theory, as articulated by Cooper and Foster [30] and Trist [32]. This perspective emphasizes that organizational outcomes are shaped by the interplay between social and technical components. Drawing on both the literature and our combined academic and practical experience—each author contributing over a decade of work in health care digital transformation—we discussed current digitalization efforts from a sociotechnical standpoint.

Each identified element, if present, was classified according to whether it had been observed in practice, research, or both, informed by both prior studies and the authors’ empirical insights. Building on this analysis, we proposed a distinction between elements that support (“on-track”) or hinder (“off-track”) health care digitalization across each sociotechnical dimension. The initial classification of these elements was conducted independently by each author, followed by a collective discussion to reach alignment. In cases where agreement could not be achieved, the majority view prevailed, with no possibility of a tie due to the odd number of contributors. This reflective process led to the development of RPs intended for further exploration, refinement, and empirical validation.

This structured approach enables a clear understanding of how social and technical factors jointly influence digital transformation in health care, guiding future empirical investigations and managerial decision-making. While these propositions were organized according to individual STS dimensions to enhance clarity and analytical precision, it is important to note that health care organizations are more likely to succeed when these dimensions are advanced simultaneously. We also recognize that interdependencies and overlaps may exist among these dimensions, although these relationships were not explicitly examined in this study.

## Discussion

### Research Propositions

Drawing on extensive debate among authors, this section discusses health care digital transformation through the lens of STS. Following Challenger and Clegg [101] and Davis et al [100], the analysis adopts the 6 STS dimensions (ie, people, culture, goals, processes/procedures, infrastructure, and technology) as a framework. Table 2 summarizes the analysis, which led to the development of interpretive propositions derived from debate rather than empirically tested relationships within this paper.

**Table 2.** Analysis of health care digital transformation according to the sociotechnical systems (STS) dimensions.

STS dimension	On-track elements	Off-track elements
Social		
People	Patient and staff engagement	Lack of technical skills Resistance to change Burnout from digital overload
Culture	Innovation-friendly culture Digitalization done more responsibly	Hierarchical decision-making Siloed departments
Goals	Digital health ecosystem pilots Alignment with value-based care	Misaligned incentives Short-term, cost-oriented focus instead of long-term benefits
Technical		
Processes/procedures	Standardized workflows Lean and agile methods Scaling remote patient monitoring	Inadequate change management Poor data governance Fragile trust frameworks
Infrastructure	Increasing investments Multiple growth options	Poor cybersecurity measures Legacy information technology stacks High implementation and maintenance costs
Technology	Artificial intelligence and predictive analytics Upgrades in “digital front doors”	Siloed interoperable systems Artificial intelligence hype

### Health Care Digital Transformation and People

A critical success factor for health care digital transformation is people, as both patients and staff define how technologies are incorporated into treatments and administrative processes, shaping more effective health systems [20,91,111]. Regardless of the technology, users must understand its implications and adapt their behaviors accordingly, since participation and shared responsibility enhance adoption, whereas imposition often leads to resistance [89,112,113]. For instance, real-time data from sensors and cloud systems only yield benefits if integrated into medical decision-making [114], rather than being ignored by unchanged operational procedures, which may lead to underused investments and frustrated outcomes.

Technical skills are also essential [115]. Many health organizations face competence gaps that hinder digital transformation and increase perceptions of loss during change processes [116]. Although digital literacy and data analytics capabilities have grown, they remain underdeveloped among clinicians, especially frontline workers [117,118]. These gaps, combined with resistance to change, limit adoption and may intensify feelings of overload and insecurity [119]. For instance, some physicians fear that digital technologies interfere with autonomy or serve as tools of managerial control [120]. However, greater exposure to and understanding of digital tools tend to increase acceptance, as shown by Pan et al [89]. Still, many clinicians may not have the time or resources to build digital competencies, and outdated systems often create fragmented user experiences [121].

Overall, both our practical and academic observations align with prior research, confirming the central role of people in health care digital transformation. Despite initiatives to increasingly engage patients and staff [63,122], barriers such as insufficient technical skills (possibly measured by a skill development matrix or digital literacy), resistance to change, and burnout from digital overload remain significant. Thus, assessing whether health care digital transformation is on the right track from a people perspective requires considering both enablers and inhibitors, as reflected in the following propositions:

P_1A_: Patient and staff engagement encourages health care digital transformation (on-track).

P_1B_: Lack of technical skills, resistance to change, and burnout from digital overload discourage health care digital transformation (off-track).

### Health Care Digital Transformation and Culture

Organizational culture refers to the shared values, beliefs, and assumptions that guide members’ behaviors and decision-making [123,124]. It is shaped by leadership, organizational history, and the socioeconomic context [125], as well as by previous experiences with change, depending on whether it was approached with honesty or manipulation [112,113]. In the digital transformation context, culture is widely recognized as a critical determinant of success [126-128]. Several studies emphasize the relevance of design principles (eg, interoperability, decentralization, and service orientation) to support the desired cultural behaviors for digital transformation [129-131].

In health systems, the cultural component extends beyond organizational boundaries, as societies differ in how they approach and promote health care [132] and its digitalization [48]. Although digital technologies can enhance access, they may exacerbate inequalities by excluding populations such as older adults, rural residents, and socioeconomically disadvantaged groups with limited digital literacy or connectivity [133]. If not addressed, the digital divide transforms a tool of inclusion into one of stratification. Many digital initiatives fail to consider cultural and contextual diversity, producing solutions misaligned with patients’ capabilities and expectations [134]. Bridging this divide requires adaptive interface design, embedded literacy programs, and an inclusive culture that values participation and diversity.

Despite strict medical and regulatory standards, innovation has historically been intrinsic to health care [135]. As technologies have evolved, health care systems have become testing grounds for new solutions, influencing both clinical and administrative processes [15,136]. This tradition has fostered an innovation-friendly culture, which somewhat favors digitalization. At the same time, increasing demands for compliance, safety, quality, and accountability [137-139] have driven more responsible and cautious digitalization [140].

However, hierarchical and siloed structures still dominate most health organizations, undermining collaboration and knowledge sharing [141]. Since effective digitalization requires coordinated efforts among clinicians, digital experts, administrators, and external partners, a culture that promotes interdisciplinary collaboration is essential but not yet widespread [142]. Therefore, the following propositions concerning the cultural perspective are suggested:

P_2A_: An innovation-friendly and responsible digitalization culture encourages health care digital transformation (on-track).

P_2B_: Hierarchical decision-making and siloed departments discourage health care digital transformation (off-track).

### Health Care Digital Transformation and Goals

STS theory establishes that joint optimization of social and technical aspects is essential for achieving a system’s goals [30]. Therefore, clearly defining strategic objectives is fundamental in digital transformation [143,144]. PricewaterhouseCoopers [145] reported that only 53% of organizations have a long-term digital transformation roadmap integrating processes, technologies, and capabilities, implying that nearly half may initiate digitalization without fully defined goals. Boston Consulting Group [146] further reported that only 30% of organizations meet their digital transformation goals, pointing to widespread issues in alignment, planning, and execution. In health care, McKinsey & Company [147] found that 75% of executives prioritize digitalization, yet planning remains insufficient, often favoring short-term, cost-oriented goals over long-term benefits. Regulatory frameworks also lag behind iterative digital health innovations, with start-ups facing ambiguous requirements and established actors encountering costly compliance obligations, thereby constraining innovation or deployment [148]. These misalignments can produce inconsistent incentives and confusion regarding health care system objectives [149,150].

On the other hand, according to WHO [151], initiatives such as the Country Health Information Systems and Data Use (CHISU) program demonstrate positive potential. Country Health Information Systems and Data Use strengthens countries’ capacity to manage high-quality health information systems, promotes evidence-based decision-making through cross-governmental working groups and district data use meetings, and supports the transition from paper registers to online visualizations. An example is the Indonesian digital health ecosystem, which integrates multiple health applications to provide data connectivity, analysis, and services aligned with value-based care [151]. While further initiatives are needed to advance health care digital transformation, existing pilots illustrate how digital ecosystems can enhance quality and efficiency at lower costs [79,152].

Based on these arguments, we formulate the following propositions associated with the goals dimension:

P_3A_: Digital health ecosystem pilots and alignment with value-based care encourage health care digital transformation (on track).

P_3B_: Misalignment of incentives and a short-term, cost-oriented focus, rather than long-term benefits, discourages health care digital transformation (off-track).

### Health Care Digital Transformation and Processes/Procedures

Health care digital transformation has evolved from the simple digitization of analog workflows to a structural reshaping of care delivery. Far from being a technological add-on, it has redefined the operational logic of health care, influencing the timing, scope, and structure of clinical and administrative routines [153]. The shift toward a digital-first approach emphasizes automation, integration, and patient self-service, making digital interactions central to care delivery [154]. Key enabling technologies, such as EHRs, provide longitudinal access to patient data, diagnostics, and treatments [155], enhancing coordination but also introducing new operational challenges [156]. Automation tools, such as robotic process automation, have reduced clerical workloads and increased data accuracy, while telemedicine and remote patient monitoring extend the boundaries of care, allowing early intervention and chronic disease management through wearables and continuous data transmission [157-159].

These capabilities have driven a paradigm shift away from episodic to preventive and personalized care. Predictive analytics and AI-assisted diagnostics help identify clinical deterioration and anticipate complications, transforming how risk is managed [160]. Patients are now active participants in decentralized health networks, accessing online portals, receiving automated alerts, and contributing to data-driven treatment plans. This evolution has been linked to higher patient satisfaction, fewer missed appointments, and gains in efficiency, including reduced documentation time and shorter check-in and discharge processes [161].

However, implementation challenges persist due to health care’s sociotechnical complexity. Digitalization intended to streamline workflows often results in fragmented digital ecosystems requiring multiple logins and interfaces, with inconsistent data structures [152]. Discrepancies between system design and clinical practice have led to frequent workarounds that undermine data integrity and safety [162]. Furthermore, alert fatigue, cognitive overload, and documentation burden caused by poorly integrated EHRs are cited as contributors to burnout, ultimately offsetting the anticipated gains in efficiency [163].

Thus, the success of digital transformation in health care processes depends less on technological novelty and more on alignment with real-world clinical practices. Effective digitalization requires co-designing systems with frontline users, establishing strong data governance and cybersecurity frameworks, embedding equity and usability in design, and addressing organizational frictions that limit adoption. Although Lean and Agile methods have supported workflow standardization and continuous improvement [164,165], many systems still face inadequate change management, weak data governance, and fragile trust frameworks. To further explore these dynamics, the following propositions concerning the processes/procedures perspective are advanced:

P_4A_: Standardized workflows, Lean and Agile methods, and the scaling of remote patient monitoring encourage health care digital transformation (on-track).

P_4B_: Inadequate change management, poor data governance, and fragile trust frameworks discourage health care digital transformation (off-track).

### Health Care Digital Transformation and Infrastructure

Even when core health care digital transformation elements of access, quality, efficiency, and equity are in place, their related processes increasingly rely on digital infrastructures [166]. Infrastructure enables transformation through physical components (eg, sensors and microcontrollers capturing data for real time or postprocessing), connectivity (eg, linking products and services to network devices and servers), and digital layers (eg, storage, analysis, and processing of large-scale data) [167,168]. Key components include data centers, cloud platforms, communication networks (5G and fiber optics), and software applications, which support data management, automation, connectivity, and service delivery. Such infrastructure is vital for innovation, economic growth, and the broader digitalization of society [169]. Global health care digital transformation investment has surpassed US $1.3 trillion, with an annual growth rate of 10.4%, and an additional US $1.5 trillion is expected in the United States alone in the coming years [151]. These investments allow health systems to address major challenges and prioritize high-impact areas [19,146], enhancing market position and enabling multiple growth options [147].

However, several factors may hinder digitalization. Security and privacy remain critical, as breaches undermine trust and regulatory compliance [170]. User-facing applications often implement measures that are either too weak or too complex, reducing effectiveness [171]. Legacy infrastructures in older hospitals (>20 y) further constrain transformation, whereas newer facilities with modern infrastructure face fewer barriers [48]. In the United Kingdom National Health Service, outdated systems force manual workarounds, creating delays and patient safety risks. Despite decreasing costs and greater accessibility [136], implementation still requires high capital expenditure, which is particularly challenging for smaller organizations (<150 inpatient beds) and those in emerging economies reliant on imported components [48,172].

Given the aforementioned, the following propositions concerning the infrastructure perspective are presented:

P_5A_: Increasing investments and multiple growth options encourage health care digital transformation (on-track).

P_5B_: Poor cybersecurity measures, legacy information technology stacks, and high implementation and maintenance costs discourage health care digital transformation (off-track).

### Health Care Digital Transformation and Technology

Technological innovations are among the primary drivers of health care digital transformation [173]. Their impact has been evident during disruptive events such as the COVID-19 pandemic [20,49] and continues thereafter. For instance, telehealth visits increased from 840,000 in 2019 to 52.7 million in 2020, with 45% of patients expressing willingness to continue using telehealth postpandemic [147]. Digital technologies, including AI, traditional machine learning, and deep learning, are expected to generate up to US $360 billion in health care savings, with virtual health and digital front doors (ie, the set of digital channels and tools that patients use as their first point of contact with a health care provider) cited by 70% of professionals as having the greatest impact [147]. As health care advances toward more sophisticated applications (eg, generative AI for clinical documentation, virtual command centers for patient monitoring, and AI-assisted surgical decision support), the focus shifts from innovation to effective integration [157,174]. Therefore, developments in AI, predictive analytics, and digital front-door upgrades may significantly accelerate health care digital transformation.

However, several technological barriers persist. Data interoperability remains a structural bottleneck: health data are often siloed within proprietary systems, limiting AI and analytics applications across the care continuum. Heterogeneous data standards, inconsistent terminologies, and vendor lock-in hinder timely information access and affect both clinical decision-making and public health surveillance [155,175]. These technical barriers are reinforced by organizational fragmentation and resistance to shared data governance [136,150], highlighting interoperability as a sociotechnical rather than purely technical challenge. Despite widespread recognition of AI’s potential, 20% of health care executives do not plan to invest in it within 2 years, jeopardizing value creation in critical areas such as referral management, operating room optimization, and care gap closure [147].

These limitations reveal the inadequacy of viewing health systems as easily programmable entities and instead emphasize the need for a sociotechnical perspective, where digital technologies are embedded within complex systems shaped by professional norms, organizational routines, and institutional structures [176]. To better investigate how technology-oriented elements may impact health care digital transformation, we raise the following propositions:

P_6A_: the level of AI and predictive analytics development and upgrades in “digital front doors” encourage health care digital transformation (on-track).

P_6B_: siloed interoperable systems and the AI hype discourage health care digital transformation (off-track).

## Final Remarks and Future Research Opportunities

The developed RPs enable the identification of on- and off-track elements and contribute to a more systematic understanding of health care digitalization. Viewing health care digitalization through an STS lens allows for a holistic comprehension of its complexity, although the interrelations among social and technical dimensions and potential trade-offs (eg, legacy infrastructure may exacerbate staff burnout, suggesting isolated technical upgrades risk suboptimal outcomes) were beyond the scope of this discussion. This strengthens theoretical coherence without overextending scope.

Our work suggests that, despite cross-country differences in health systems, the elements that encourage or hinder health care digitalization can be delineated. This supports the establishment of more effective government policies, the design of private initiatives, and the adjustment of regulatory frameworks. From a research perspective, interdisciplinary avenues emerge:

1. *Establishing open innovation for promoting health care digitalization*. Collaborative partnerships (eg, joint ventures and alliances) have proven valuable in other industries for combining capabilities, accelerating time-to-market, and achieving scale and efficiency. In health care, open innovation can leverage on-track elements such as an “innovation-friendly culture” and “digital health ecosystem pilots,” while mitigating off-track ones, such as a “lack of technical skills” and “siloed interoperable systems.” Future studies could explore mechanisms that facilitate this balance and promote cross-sector collaboration.
2. *Adapting health systems’ operations to the digital era*. Digitalization requires operational changes in structure (eg, cross-functional and empowered teams), talent (new skill sets and dedicated digital roles), work practices (patient-centric models), and technology (modular, cloud-based architectures). Although this adaptation is complex and time-consuming, it can be catalyzed by existing on-track elements such as “patient and staff engagement” and “lean and agile methods.” Additional research could address off-track elements such as “resistance to change” and “inadequate change management.”
3. *Carefully developing AI-based solutions for health care*. AI has transformative potential across health care functions, from clinical operations to corporate management, but it also introduces ethical, legal, and privacy concerns. The development of AI-based solutions must integrate risk management and legal oversight alongside technical teams, guided by a clear prioritization of patient safety and accountability. Future research could deepen the understanding of AI’s real-world benefits, distinguishing evidence-based progress from technological hype.
4. *Understanding the effects of socioeconomic and political aspects on health care digitalization*. Different socioeconomic contexts may either hinder or favor a more extensive digitalization of health care. Issues such as infrastructure and capital expenditure capacity, which are necessary mainly from a technical point of view, may vary significantly, raising additional challenges to successful health care digitalization. Government policies can play a relevant role, mitigating barriers and catalyzing digitalization efforts. Further studies could encompass these aspects and check their relevance for digitizing both public and private health systems.

From a practical standpoint, health systems pursuing digitalization should simultaneously strengthen social and technical dimensions, reinforcing on-track elements while redirecting off-track ones. Given the contextual nature of health care delivery, digital transformation must be tailored to each system’s specific needs, adding complexity to efforts by policymakers, organizations, academia, and regulators. This study contributes by mapping the sociotechnical factors influencing health care digitalization and by drawing practitioners’ attention to potential barriers, enablers, and strategies for achieving digital progress.

Regarding limitations, since this is a viewpoint paper, it does not claim systematic empirical verification of all proposed relationships. Instead, the formulated RPs should be used as input for future studies, so that they can be tested and validated. Additionally, STS theory states that the joint optimization of social and technical elements leads to successful approaches and greater performance. We discussed social and technical elements separately disregarding their interrelationship. Further research should be conducted to verify how such an interaction can either favor or hinder health care digitalization. Furthermore, our work mainly discusses health care digitalization based on evidence collected in major economies, where data and literature tend to be more available. However, regions such as Africa present a unique context in which fragmentation, limited scalability, and unequal investments pose additional challenges [177,178]. More research should be conducted to properly explore health care digitalization from a more global perspective. Finally, an effective digital transformation may be subject to other aspects beyond the ones discussed here. The interests of stakeholders such as lobby agencies, government, and regulatory institutions might influence the speed of digitalization changes, raising additional challenges. Research on these aspects should be carried out to enhance the holistic view of the implications of digitalization.

## Abbreviations

AI: artificial intelligence
EHR: electronic health record
EMR: electronic medical record
GAI: generative artificial intelligence
RP: research proposition
WHO: World Health Organization
